# Men in Nursing

**DOI:** 10.17795/nmsjournal17987

**Published:** 2014-04-17

**Authors:** Negin Masoudi Alavi

**Affiliations:** 1 Trauma Nursing Research Center, Kashan University of Medical Sciences, Kashan, IR Iran

**Keywords:** Male, Healthcare, Nursing

Dear Editor,

The December issue of Nursing and Midwifery Studies Journal contains an interesting article about factors influencing men entering the nursing profession, and provides an understanding of the challenges they face. The authors believe that although the number of men entering the nursing profession is on the rise, yet there are still many challenges in front of our male colleagues in this female dominated profession (1). Some may question the caring capabilities of male nurses (2). Others may believe that nursing is not a suitable career choice for men (3). Most of the problems come from stereotypical images around nursing and also around gender roles. A study in the United States found that in television series, the male nurses were often subject to questions about their choice of career, masculinity and sexuality and their role is usually minor or a source of comedy (4). I as a female nurse have been frequently confronted with this stereotypical image around my gender. The belief, that women are not good drivers, and studying engineering is not appropriate for women and so many other bizarre cultural and sometimes universal beliefs are familiar to us. So we as a potential victim of these unrealistic views should understand our male colleagues better and more actively support them.

Firstly, we should accept that men are a reality in nursing. In England 10% of nurses are men (1). In Iran, about 23% of nurses were men in 2006 (5). Secondly, we should believe that men could bring a new fuel and insight and energy to the profession. Thirdly, we have to reconsider some traditional beliefs about gender issues. Male participants in a qualitative study revealed that gender-based stereotypes contributed to their job dissatisfaction, and on the other hand providing care to patients and making a difference were personal rewards that motivate them to stay in the profession (6). Another study showed that male nurses were highly focused on patient care and transformational potential for personal fulfillment (7). You can see that the picture that some have, could not be farther from reality. I remember a dialogue from a movie where a male character was asking his female colleagues why men die more from heart attacks and he answered himself by saying because they have a suffering heart. Today, educational developments, changes in traditional societies and promotion of gender equality in education and employment have opened doors to both males and females to play their unbiased roles in healthcare services and in their communities according to their interests and their abilities. I hope that male nurses will increasingly create their personal core values in healthcare teams. In this challenge they should see their female colleagues on their side.
